# Olanzapine Versus Aprepitant for the Prevention of Chemotherapy-Induced Nausea and Vomiting: A Systematic Review and Meta-Analysis

**DOI:** 10.7759/cureus.83118

**Published:** 2025-04-28

**Authors:** Indrani Sarma, Sukainnya Buragohain, Joonmoni Lahon, Priyotosh Banerjee, Subodh Kumar, Rituparna Chetia, Pakesh Baishya, Dibyajyoti Saikia

**Affiliations:** 1 Pharmacology, All India Institute of Medical Sciences, Guwahati, Guwahati, IND; 2 Pharmacology, ICARE Institute of Medical Sciences and Research (IIMSAR), Haldia, IND; 3 Pharmacology, All India Institute of Medical Sciences, Deoghar, Deoghar, IND; 4 Medical Oncology, All India Institute of Medical Sciences, Guwahati, Guwahati, IND; 5 Pathology, All India Institute of Medical Sciences, Guwahati, Guwahati, IND

**Keywords:** antiemetics, aprepitant, cinv, meta-analysis, nausea, olanzapine, somnolence, systematic review, vomiting

## Abstract

Cancer care faces challenges with chemotherapy-induced nausea and vomiting (CINV). Olanzapine and aprepitant, alone or with traditional antiemetics, promise CINV prevention. Their mechanisms target neurotransmitters, providing better control with manageable side effects. This study aims to rigorously review the efficacy and safety of olanzapine versus aprepitant in preventing CINV.

Randomized clinical trials comparing olanzapine versus aprepitant in preventing CINV were selected following Preferred Reporting Items for Systematic Reviews and Meta-Analysis (PRISMA) 2020 guidelines. A comprehensive literature search was conducted in various databases and registries through March 21, 2024. Quality assessment utilized Cochrane's 'Risk of Bias tool (RoB2)', and Review Manager 5.4.1 synthesized results using a random effect model. The main outcomes focused on odds ratios (ORs) for complete response (CR) in acute, delayed, and overall phases. Safety was assessed from trial descriptions. The certainty of evidence was assessed using GRADE Pro.

Two hundred and eighty-two records were identified initially, yielding eight eligible RCTs with a total of 1056 participants after screening. All studies targeted CINV in adults undergoing highly emetogenic chemotherapy. In the acute phase, both drugs demonstrated similar efficacy in nausea and vomiting control (OR = 1.01, 95% CI = 0.63,1.62). During the delayed phase, no significant difference was observed (OR = 0.81, 95% CI = 0.62,1.04). In the overall phase, olanzapine exhibited slightly better nausea control than aprepitant, with statistical significance (OR = 0.76, 95% CI = 0.59,0.99). Emetic control was comparable across treatment arms (OR 0.93, 95 % CI 0.70-1.24).

Olanzapine provided a clinically meaningful reduction in nausea. However, the sedation caused by olanzapine, which is significantly higher than that caused by aprepitant, can impair daily functioning, diminish treatment adherence, and increase fall risk.

## Introduction and background

Chemotherapy-induced nausea and vomiting (CINV) remain significant challenges in cancer treatment, impacting patients' quality of life and treatment adherence [1]. Despite advancements in antiemetic therapy, a substantial proportion of patients still experience CINV, leading to distress and compromised well-being. Other adverse drug reactions caused by chemotherapy include hair fall, leukopenia, diarrhea, and gastritis. Clinically, CINV is classified as acute, delayed, anticipatory, breakthrough, or refractory, each demanding tailored prophylaxis or rescue. Current guidelines recommend quadruple prophylaxis, an NK₁ receptor antagonist plus a 5-HT₃ antagonist, dexamethasone, and low-dose olanzapine for highly emetogenic regimens, with triple or olanzapine-based combinations for moderate risk protocols [2].

Despite these advances, 70-80 % of adults still experience some degree of CINV during chemotherapy, and nausea/vomiting alone accounts for roughly one-quarter of all documented chemotherapy adverse drug reactions [1]. In recent years, there has been a growing interest in the use of prophylactic antiemetics, particularly regimens containing olanzapine and aprepitant, for the prevention of CINV [2]. These medications, either alone or in combination with traditional antiemetics, have shown promising results in clinical trials, offering improved control of acute (the first 24 h after the start of chemotherapy), delay (24 to 120 h after chemotherapy), and overall (0 to 120 h after chemotherapy) phases of CINV. This study aims to explore the rationale behind the utilization of olanzapine and aprepitant in CINV prophylaxis and the emerging evidence supporting their efficacy and safety profiles. Understanding the role of these agents in CINV management is crucial for optimizing treatment strategies and enhancing the overall well-being of cancer patients undergoing chemotherapy.

Initially developed as an antipsychotic medication, olanzapine's off-label use in CINV management stems from its potent antiemetic properties [3]. Olanzapine acts by antagonizing multiple neurotransmitter receptors, including dopamine, serotonin, histamine, and muscarinic receptors, within the central nervous system. This broad-spectrum activity enables olanzapine to effectively address both acute and delayed phases of CINV.

Clinical trials have demonstrated the efficacy of olanzapine in CINV, showcasing its superiority over traditional antiemetic regimens [4]. Olanzapine's ability to synergize with existing antiemetic agents further enhances its utility in comprehensive symptom control, positioning it as a promising option for CINV management.

Aprepitant belongs to the class of neurokinin-1 (NK-1) receptor antagonists, specifically designed to target the substance P receptor involved in the emetic pathway [5]. By inhibiting substance P binding, aprepitant blocks the transmission of signals responsible for triggering nausea and vomiting. Initially approved for use in highly emetogenic chemotherapy regimens, aprepitant has since demonstrated efficacy across various chemotherapy protocols and settings [6].

Clinical evidence supports the effectiveness of aprepitant in reducing both the incidence and severity of CINV, particularly in the acute phase [7]. Its role in preventing delayed CINV further underscores its importance in optimizing patient comfort and treatment compliance. International guidelines offer recommendations regarding the use of olanzapine and aprepitant in CINV management, acknowledging their distinct mechanisms and therapeutic benefits [8]. 

The Multinational Association of Supportive Care in Cancer (MASCC) and the European Society for Medical Oncology (ESMO) guidelines advocate for the incorporation of olanzapine into antiemetic regimens, emphasizing its efficacy in addressing both acute and delayed CINV [8]. Similarly, the American Society of Clinical Oncology (ASCO) guidelines endorse the use of aprepitant-containing regimens, particularly in patients undergoing highly emetogenic chemotherapy, to optimize CINV prophylaxis [9]. Though there are no established statutory guidelines regarding treatment of CINV in India, a multidisciplinary group of Indian oncologists, pharmacologists, and supportive-care specialists produced the “Expert Consensus on Effective Management of CINV: An Indian Perspective” in 2020, which also supports similar treatment protocols [10]. The available data claims that olanzapine and aprepitant show similar efficacy in chemotherapy patients [10].

Aprepitant-based triple therapy remains guideline-recommended for highly emetogenic chemotherapy, yet olanzapine has emerged as a low-cost alternative that several recent trials report as equally effective, especially for delayed-phase nausea. Head-to-head evidence, however, has been inconsistent. An up-to-date quantitative synthesis was therefore essential to clarify the comparative efficacy and safety of both the drugs. Previous systematic reviews either compared olanzapine against placebo or standard care, combined adult and pediatric data, or ranked multi-drug regimens in network analyses without isolating direct olanzapine-versus-aprepitant comparisons. Our review focuses exclusively on adult, parallel-group trials that directly compare olanzapine and aprepitant and gives a more clinically actionable comparison to guide antiemetic protocols.

## Review

Methods

Inclusion and Exclusion Criteria

The study has been registered in PROSPERO under CRD42024524364. Our study included published and completed randomized controlled trials of olanzapine versus Aprepitant to prevent CINV in adult cancer patients receiving chemotherapy. Aprepitant in combination with a standard antiemetic regimen was taken as control and olanzapine was added to the standard antiemetic regimen as intervention. The evaluation indicators included complete response (no vomiting and no rescue) in acute, delayed, and overall periods for the prevention of CINV and incidence of sedation.

We excluded duplicate published literature studies (literature studies with the completed trial and up-to-date data retained), studies done in different populations other than adults, those without olanzapine as a head-to-head comparator to aprepitant and studies that have other treatment regimens. Articles published in a language other than English were excluded.

Literature Retrieval Strategy

A comprehensive literature search was made in databases such as PubMed, Ovid, Cochrane, Scopus, MEDLINE, WHO Global Index Medicus, PsycINFO, and registries such as CTRI, ISRCTN, CT. gov, and ICTRP were conducted to collect published randomized controlled trials of olanzapine versus Aprepitant for the prevention of CINV.

The relevant literatures were searched from the establishment of the databases and registries to 21st March 2024. In order to search the whole literature, we carry out the retrieval mode of subject words and free words, including vomiting or nausea, olanzapine, Aprepitant, and all kinds of free words.

Literature Screening and Data Extraction

Literature screening, data extraction, and cross-checking were conducted by two researchers independently using Rayyan and Systematic Review Accelerator. In case of disagreement, consensus was reached through discussion or consultation with a third researcher. Literature scr

Screening was conducted by reading the title and abstract and further reading of the full text after excluding the apparently irrelevant literature to determine whether it was included or not.

Data extraction was done in duplicate using a structured data extraction form that includes (i) the research of the basic characteristics, including research types, article name, year, authors, published by use of chemotherapy regimens, patients with cancer types, age, gender, patients’ basic information, project for the time range, control group- and experimental group-specific regimen to prevent nausea and vomiting; (ii) key elements of bias risk assessment; and (iii) outcome indicators and outcome measurement data of concern.

The main outcomes were the odds ratios (OR) for complete response (CR, no nausea and no rescue) in the acute (0-24 h post-chemotherapy), delayed (24-120 h post-chemotherapy), and overall (0-120 h post-chemotherapy) phases. The safety endpoint was the incidence of sedation.

Quality Evaluation of Included Studies

We strictly followed Preferred Reporting Items for Systematic Reviews and Meta-Analysis (PRISMA) 2020 guidelines. Version 2 of the Cochrane risk-of-bias tool for randomized trials (RoB 2) was used independently by two investigators to assess the risk of bias in the included studies.

For individually randomized studies, we assessed RoB in five domains: (i) the randomization process; (ii) deviations from intended interventions; (iii) missing outcome data; (iv) measurement of the outcome; and (v) selection of the reported result. Domain 1 was assessed at the study level and the other domains were assessed at the result level.

For each domain, we made a judgment of high RoB, low RoB, or some concerns. We used the signaling questions and algorithm and considered whether to override the algorithm result, recording our reasons and supporting evidence.

Statistical Analysis

Meta-analysis was performed using RevMan 5.4 software, combining categorical variables with odds ratios (ORs). Confidence interval (CI) of 95% was taken for significance. Heterogeneity was assessed using the random effects model due to variation in the adult population, and sensitivity analysis was done if I² exceeded 50%. The inverse variance method and the Mantel-Haenszel method were used to pool data. The inverse variance method weights studies based on the inverse of their variance, favouring more precise estimates. The Mantel-Haenszel method adjusts for confounding factors by calculating a weighted average of odds ratios or risk ratios across strata. A p-value of less than 0.05 was considered statistically significant..

Results

Included Studies

**Figure 1:**
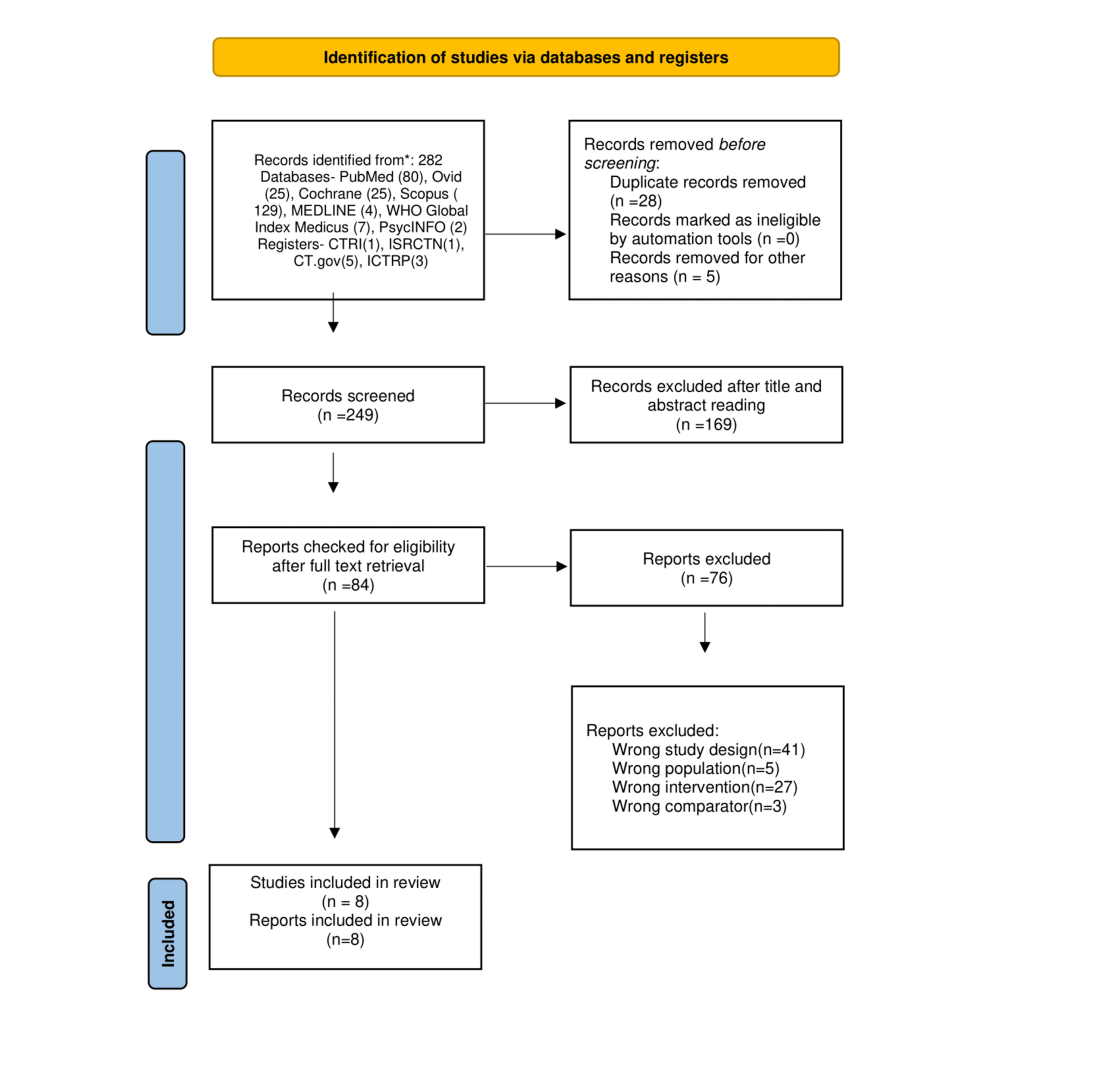
PRISMA flowchart depicting the selection process of the studies

From the initial search, 282 records were identified. After deleting 28 duplicate records and excluding five studies for being at a recruiting stage or not having reported results, a total of 249 records entered the initial screening. After initial screening through titles and abstracts, we ended up with 84 studies for full-text retrieval and analysis. Seventy-six studies were reasonably excluded. A total of eight studies have eligibility criteria for systematic evaluation. All of these studies have been published in peer-reviewed journals. The PRISMA study selection diagram is shown in Figure 1.

All included studies were randomized controlled trials and matched the eligibility criteria. All eight studies reported a CR to antiemetic therapy and defined CR as no vomiting and no rescue. A table detailing the outcome definitions has been included here as Table 1.

**Table 1:** Outcome measures of the included studies

Serial No.	Study ID	Definition of Complete Response (CR)	Assessment Method of Outcome Measures
1	Liu et al., 2022 [11]	CR refers to no vomiting symptoms and no use of rescue drugs	The functional life index nausea and vomiting (FLIE) questionnaire. Nausea score uses VAS. The occurrence of adverse events (AE) was graded according to Common Terminology Criteria v.4.0.
2	Mohammed et al., 2022 [12]	Complete response (no emesis and no rescue therapy	CTCAE grading
3	Sapkota et al., 2023 [13]	Complete response (no emesis and no rescue),	The questionnaire method was used. Grading of nausea, vomiting, and adverse effects was done using CTCAE version 4.
4	Navari et al., 2011 [14]	CR no emetic episodes and no use of rescue medication for the overall period (0–120 hours post-chemotherapy)	M.D. Anderson Symptom Inventory (MDASI) was utilized. Episodes of nausea were assessed using a visual analogue scale (VAS) from 0 to 10, with 0 indicating no nausea and 10 indicating a maximal level of nausea.
5	Ithimakin et al., 2020 [15]	CR defined as no episode of vomiting and no rescue treatment required within 120 h	Adverse events and quality of life (QOL), assessed by the Functional Living Index Emesis (FLIE). Nausea and vomiting grade according to the Common Toxicity Criteria and visual analog scale (VAS) nausea score.
6	Mukesh et al., 2023 [16]	The primary end point of the study is complete response (CR) for nausea that is no nausea in the acute, delayed and overall periods. The secondary endpoint is CR for vomiting and no use of rescue drugs in acute, delayed and overall period.	Daily episodes of nausea using a visual analogue scale (VAS) from 0 to 10. Record daily episodes of vomiting (number and time), the daily intensity of symptoms and the utilization of rescue therapy.
7	Babu et al., 2016 [17]	Complete remission rates (no emesis, no rescue)	A VAS was used to assess the intensity of nausea. All toxicities were graded using the Common Toxicity Criteria (CTC).
8	Shuang et al., 2017 [18]	CR is defined as the absence of vomiting and the absence of rescue medication	The functional life index nausea and vomiting (FLIE) questionnaire. The nausea score used VAS.

Baseline data of all the included studies are shown in Table 2.

**Table 2:** Table showing baseline characteristics of the eligible studies NI*: Not Included **: Same treatment for intervention and control groups

SL NO.	Author (Ref)	Country	Type of Chemotherapy	Total Participants	Age (Mean)	Gender	Treatment Regimen
1	Liu et al., 2022 [11]	China	Highly Emetogenic	104 (Intervention), 106 (Control)	59.26 (Intervention), 60.01 (Control)	M-64, F-40 (Intervention), M-63, F-43 (Control)	Tropisetron 5 mg IV + Dexamethasone 10 mg IV
2	Mohammed et al., 2022 [12]	India	Highly Emetogenic	77 (Both)	NI*	NI*	Palonosetron IV + Dexamethasone IV
3	Sapkota et al., 2023 [13]	Nepal	Highly Emetogenic	25 (Both)	55 (Intervention), 58 (Control)	M-12, F-13 (Intervention), M-11, F-14 (Control)	Granisetron 1 mg IV + Dexamethasone 12 mg oral
4	Navari et al., 2011 [14]	Indiana	Highly Emetogenic	121 (Intervention), 120 (Control)	63 (Intervention), 68 (Control)	M-40, F-81 (Intervention), M-37, F-83 (Control)	Dexamethasone 12 mg IV + Palonosetron 0.25 mg IV
5	Ithimakin et al., 2020 [15]	Thailand	Highly Emetogenic	46 (Intervention), 47 (Control)	52.5 (Intervention), 53.7 (Control)	M-11, F-35 (Intervention), M-9, F-38 (Control)	Ondansetron 8 mg IV + Dexamethasone 12 mg IV
6	Mukesh et al., 2023 [16]	India	Highly Emetogenic	61 (Intervention), 60 (Control)	47 (Both)*	F-61 (Intervention), F-60 (Control)	Palonosetron 0.25 mg IV + Dexamethasone 8 mg IV
7	Babu et al., 2016 [17]	India	Highly Emetogenic	50 (Both)	44.7 (Both)**	M-15, F-35 (Both)**	Palonosetron 0.25 mg IV + Dexamethasone 20 mg IV
8	Shuang et al., 2017 [18]	China	Highly Emetogenic	43 (Intervention), 44 (Control)	49.8 (Intervention), 50.3 (Control)	NI*	Tropisetron 5 mg IV + Dexamethasone 10 mg IV

Two studies [11,12] compared 5mg olanzapine to aprepitant, while six studies [13-18] compared the data between 10mg olanzapine and aprepitant. All studies used the same concurrent antiemetic regimens: a corticosteroid and a 5-HT3 receptor antagonist.

All studies are CINV for adults and include data pertaining to HEC patients. Five studies [11,13,15,17,18] reported adverse reactions, including sedation.

Quality of Included Studies

**Figure 2:**
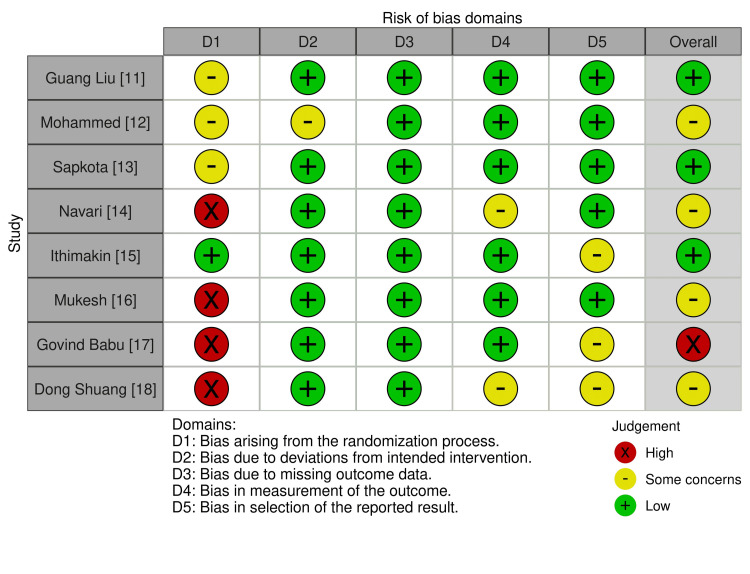
Figure depicting the risk of bias assessment of the included studies

Each bias risk assessment included in the study is reported in Figure 2. Three of the studies showed low risk of bias, while four other studies showed some concern. Only one study had a potential of high risk of bias in domain 1 among the included studies in the review. This could potentially skew the pooled estimate and increase heterogeneity.

**Figure 3:**
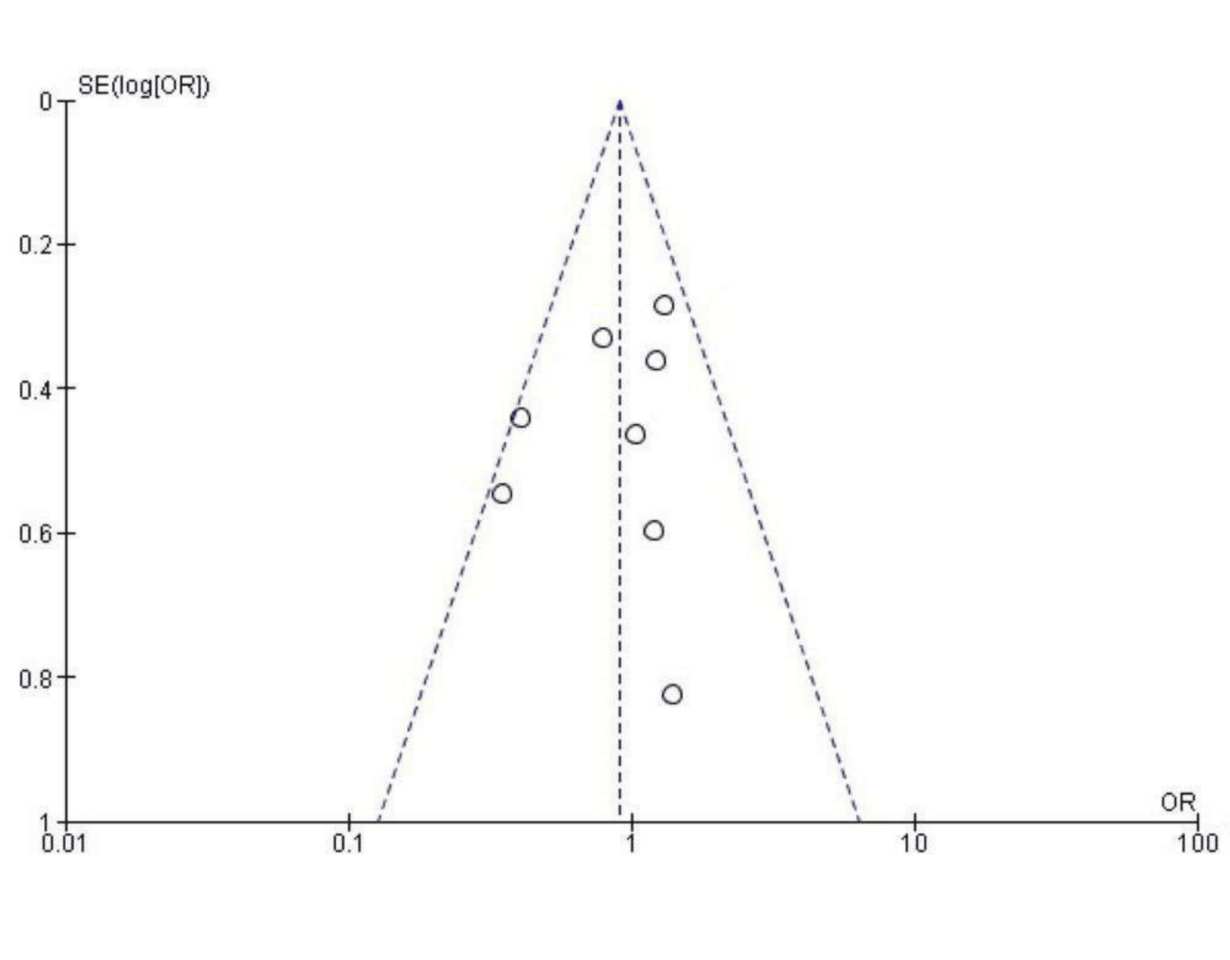
Funnel plot depicting publication bias of the literature

The funnel diagram is shown in Figure 3.

There is no obvious asymmetry, indicating that there is no obvious publication bias in this literature.

Efficacy of Olanzapine Versus Aprepitant for the Prophylaxis of CINV

**Figure 4:**
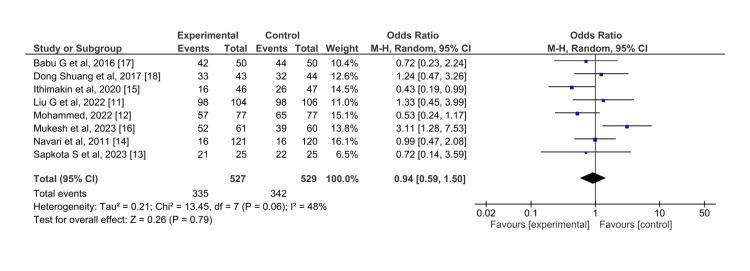
Control of nausea in the acute phase for olanzapine (experimental) versus aprepitant (control)

Acute phase: For CR in the acute phase after chemotherapy, the efficacy of olanzapine and aprepitant has no statistically significant difference. For HEC patients, olanzapine was similar in efficacy to aprepitant in case of nausea control in the acute phase (OR = 0.94, 95% CI = 0.59,1.50) (Figure 4).

The I^2^ for this effect is 48% which indicates high heterogeneity. However, the heterogeneity in this result is emerging from only one study by Mukesh et al. done in 2023 (OR=3.11) [16]. Excluding the results from the one study, the I^2^ of the remaining studies becomes 0, leading us to conclude that this study has a significant favor towards the effect of olanzapine in the control of nausea. High heterogeneity from the study by Mukesh et al. may be due to the reporting of a significant difference in the number of events in acute phase nausea events, compared to smaller differences in other phases. However, the overall pooled effect (Z= 0.03) did not register any statistically significant change between the two drugs in the case of nausea control in the acute phase with or without the particular study.

In the case of control of vomiting in the acute phase, efficacy of olanzapine and aprepitant has no statistically significant difference.

**Figure 5:**
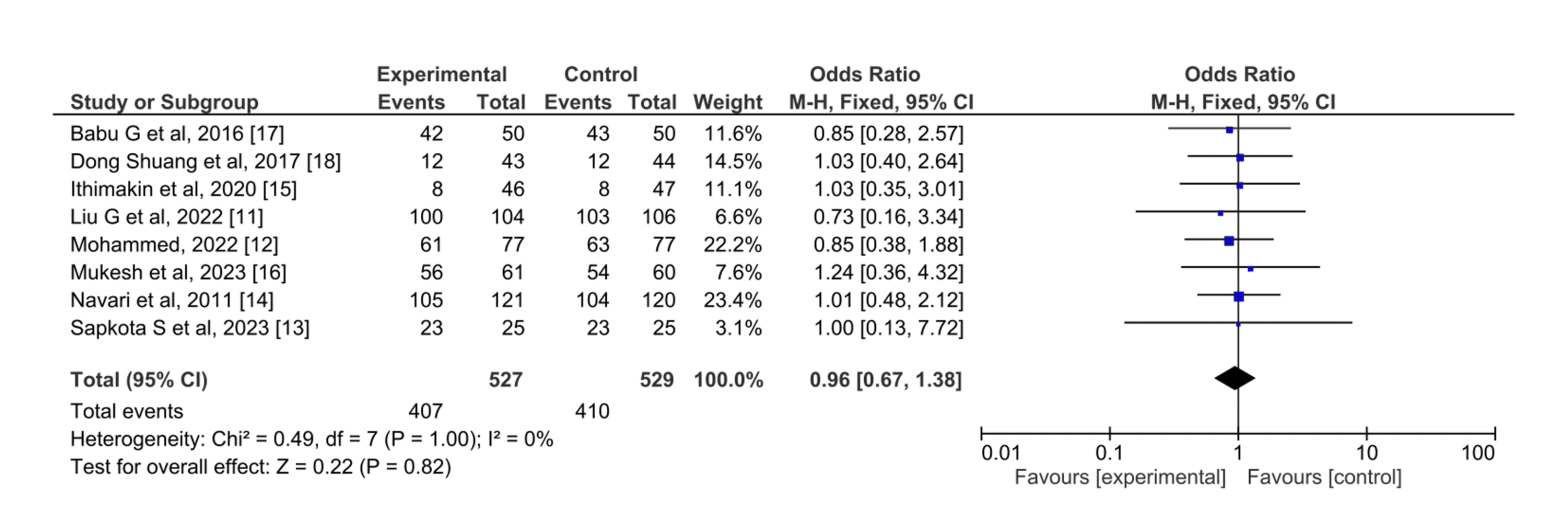
Control of vomiting in the acute phase for olanzapine (experimental) versus aprepitant (control)

Olanzapine was similar in efficacy to aprepitant in case of vomiting control in the acute phase (OR = 0.96, RR=1.64, 95% CI = 0.67,1.38) (Figure 5). The I^2^ was 0, indicating no heterogeneity across the studies and the overall effect (Z=0.22) also indicated that both drugs were equally efficacious in the control of vomiting in the acute phase.

**Figure 6:**
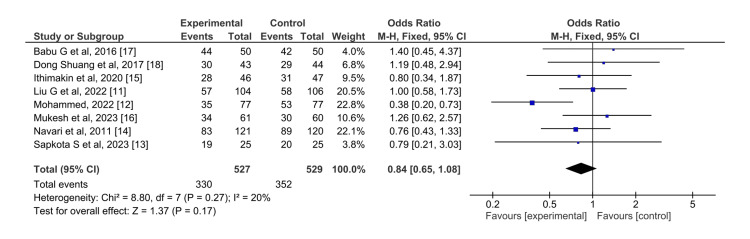
Control of nausea in the delayed phase for olanzapine (experimental) versus aprepitant (control)

Delayed phase: The effect of olanzapine and aprepitant bears no significant difference in the case of control of nausea in the delayed phase (OR = 0.84, 95% CI = 0.65,1.08) (Figure 6).

The overall effect (Z=1.37) showed no statistical significance. I^2^ for this analysis was 20%.

**Figure 7:**
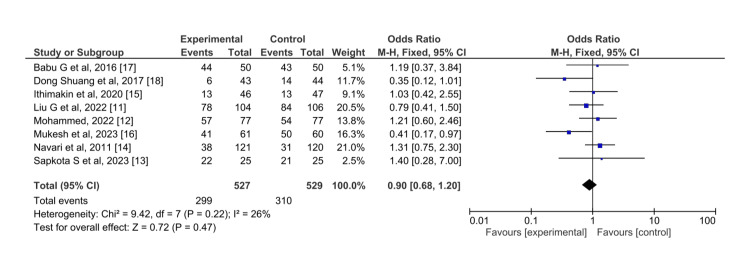
Control of vomiting in the delayed phase for olanzapine (experimental) versus aprepitant (control)

For vomiting in the delayed phase (Figure 7), olanzapine and aprepitant showed similar efficacy towards the control of vomiting in the delayed phase (OR = 0.90, 95% CI = 0.68,1.20). The overall effect was seen to be statistically insignificant (Z=0.72), and I^2^ was 26%.

In this case, the I^2^ becomes 0 on excluding the study by Mukesh et al. done in 2023 (OR= 0.41) [16]. However, the pooled effect (Z=0.72) showed no statistical difference between the drug effects.

**Figure 8:**
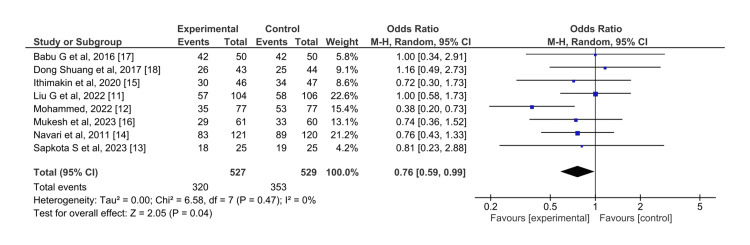
Control of nausea in the overall phase for olanzapine (experimental) versus aprepitant (control)

Overall phase: For CR in the overall phase, olanzapine is shown to have a slightly statistically significant better control on nausea (p=0.04) than aprepitant (OR=0.76, 95%CI=0.59,0.99) (Figure 8). The I^2^ is 0 here, and the pooled effect is found to be statistically significant (Z=2.05).

**Figure 9:**
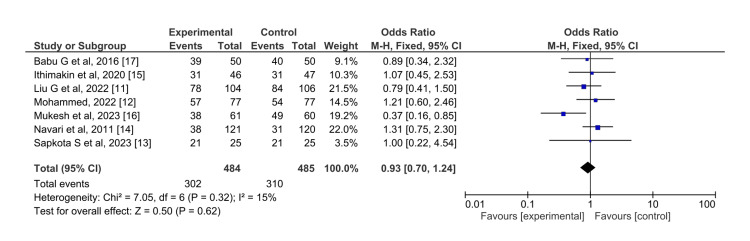
Control of vomiting in the overall phase for olanzapine (experimental) versus aprepitant (control)

For control of vomiting in the overall phase (Figure 9), the difference in efficacy of olanzapine and aprepitant had no statistically significant difference (OR=0.93, 95%CI= 0.70,1.24). The I^2^ for this result is 15% and the overall pooled effect (Z=0.50) did not show any statistically significant difference.

Safety of Olanzapine Versus Aprepitant for the Prophylaxis of CINV

**Figure 10:**
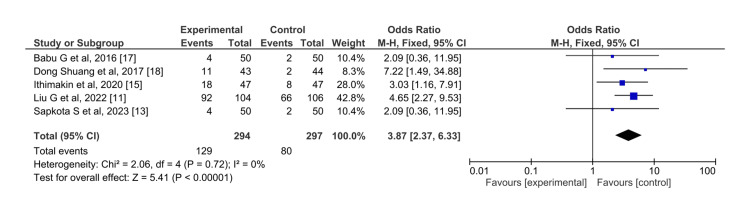
Incidence of somnolence for olanzapine (experimental) versus aprepitant (control)

Somnolence: The sedation of the olanzapine groups was statistically significant than that of the aprepitant group (P<0.00001) (Figure 10).

The incidence of somnolence was higher in the olanzapine group (OR=3.87, 95%CI=2.37,6.33). The I^2^ in this result was 0 and the overall effect showed statistically significant results (Z=5.41). A summarized table showing pooled ORs has been added for quick reference in Table 3. Subgroup analysis was not attempted.

**Table 3:** Summary table showing pooled ORs *Adverse-effect analysis was driven mainly by sedation/somnolence events. All pooled estimates showed low heterogeneity (I² ≤ 26 %).

Outcome & Phase	Pooled OR (95 % CI)	P-value	Interpretation
Acute-phase nausea	0.94 (0.59 – 1.50)	0.79	No difference
Acute-phase vomiting	0.96 (0.67 – 1.38)	0.82	No difference
Delayed-phase nausea	0.84 (0.65 – 1.08)	0.17	No difference
Delayed-phase vomiting	0.90 (0.68 – 1.20)	0.47	No difference
Overall nausea	0.76 (0.59 – 0.99)	0.04	Moderate but significant reduction with olanzapine
Overall vomiting	0.93 (0.70 – 1.24)	0.62	No difference
Any adverse effect*	3.87 (2.37 – 6.33)	< 0.00001	Significantly more adverse events with olanzapine

GRADE

Overall evidence was qualified using GRADE Pro for the randomized controlled trials. Summary of findings showed that while most information is from low or unclear risk of bias, the potential limitations are unlikely to lower confidence in the effect estimate. The effect estimate of all studies is at or near the line of no effect, the studies may be considered precise for the outcome of clinical equivalence. The GRADE Pro table is shown in Table 4.

**Table 4:** Olanzapine compared to aprepitant for CINV certainty of evidence ᵇ: Most information is from low or unclear risk of bias; the potential limitations are unlikely to lower confidence in effect estimate. ᶜ: 95% CI of pooled estimate is crossing line of no effect. ᵈ: 95% CI of pooled estimate is close to line of no effect. The effect estimate of all studies is at or near line of no effect, the studies may be considered precise for the outcome of clinical equivalence. ᵉ: The effect estimate from three studies favors control while three studies favoring intervention remaining two are close to line of no effect.

Outcome	Participants (studies) Follow-up	Risk of Bias	Inconsistency	Indirectness	Imprecision	Publication Bias	Overall Certainty of Evidence	Study Event Rates (%)	Relative Effect (95% CI)	Risk with Aprepitant	Risk Difference with Olanzapine
Acute-phase nausea	935 (8 RCTs)	Not seriousᵇ	Not seriousᵇ	Not serious	Seriousᶜ	None	⨁⨁⨁◯ Moderate	Aprepitant: 265/469 (56.5%) Olanzapine: 249/466 (53.4%)	OR 0.81 (0.57 to 1.15)	565 per 1,000	52 fewer per 1,000 (from 140 fewer to 34 more)
Acute-phase vomiting	1056 (8 RCTs)	Not seriousᵇ	Not seriousᵇ	Not serious	Seriousᶜ	None	⨁⨁⨁◯ Moderate	Aprepitant: 410/529 (77.5%) Olanzapine: 407/527 (77.2%)	OR 0.92 (0.67 to 1.38)	775 per 1,000	7 fewer per 1,000 (from 77 fewer to 51 more)
Delayed-phase nausea	1056 (8 RCTs)	Not seriousᵇ	Not seriousᵇ	Not serious	Seriousᶜ	None	⨁⨁⨁◯ Moderate	Aprepitant: 318/529 (60.1%) Olanzapine: 292/527 (55.4%)	OR 0.81 (0.61 to 1.08)	601 per 1,000	51 fewer per 1,000 (from 122 fewer to 18 more)
Delayed-phase vomiting	1056 (8 RCTs)	Not seriousᵇ	Not seriousᵇ	Not serious	Very seriousᵉ	None	⨁⨁◯◯ Low	Aprepitant: 310/529 (58.6%) Olanzapine: 299/527 (56.7%)	OR 0.88 (0.63 to 1.25)	586 per 1,000	31 fewer per 1,000 (from 115 fewer to 53 more)
Overall nausea	1056 (8 RCTs)	Not seriousᵇ	Not seriousᵇ	Not serious	None	None	⨁⨁⨁⨁ High	Aprepitant: 319/529 (60.3%) Olanzapine: 286/527 (54.3%)	OR 0.76 (0.59 to 0.99)	603 per 1,000	67 fewer per 1,000 (from 130 fewer to 2 fewer)
Overall vomiting	969 (7 RCTs)	Not seriousᵇ	Not seriousᵇ	Not serious	Seriousᶜ	None	⨁⨁⨁◯ Moderate	Aprepitant: 310/485 (63.9%) Olanzapine: 302/484 (62.4%)	OR 0.92 (0.67 to 1.27)	639 per 1,000	19 fewer per 1,000 (from 96 fewer to 53 more)

Discussion

In evaluating the primary objective of our study, we analyzed the efficacy of olanzapine compared to aprepitant for the prophylaxis of CINV. Statistical analyses indicated no significant difference between the two treatments during the acute phase for both nausea and vomiting control, aligning with findings by Vaid et al. [10]. This suggests that either drug could be reasonably selected for acute CINV management based on efficacy.

Interestingly, a study by Mukesh et al. [16] conducted in 2023 demonstrated a significant preference for olanzapine’s effect on nausea control. However, excluding this study nullified the heterogeneity among the remaining studies, indicating its potential influence on the overall heterogeneity observed.

In the delayed phase, no significant difference between olanzapine and aprepitant was observed for controlling either nausea or vomiting. Minimal heterogeneity further supports the lack of distinction between the drugs’ efficacy over a prolonged period post-chemotherapy, similar to results published by Liu et al. [11].

Contrastingly, during the overall phase of treatment, olanzapine showed a slight, yet statistically significant, greater efficacy in controlling nausea. This highlights olanzapine’s potential to provide better long-term relief from nausea, a common and distressing symptom for patients undergoing chemotherapy. For vomiting control, however, both treatments exhibited comparable efficacy.

Regarding safety, our study identified a significantly higher incidence of somnolence in patients treated with olanzapine compared to those treated with aprepitant. This aligns with the study by Rao et al. [1], which show that sedation is a known adverse effect associated with olanzapine, and it is an important consideration for clinicians when prescribing CINV prophylaxis. The absence of heterogeneity in this finding indicates a consistent outcome across studies, further validating the increased sedative effects of olanzapine.

Recent systematic reviews and meta-analyses reinforce these findings. Chow et al. [19] found that regimens containing olanzapine were significantly more effective in several efficacy endpoints in the prophylaxis setting, especially at a 10 mg dose. Similarly, Yoodee et al. [20] reported that both 5 mg and 10 mg doses of Olanzapine significantly improved control of CINV in the delayed and overall phases, although the 10 mg dose was linked to higher rates of somnolence. Additionally, Abe et al. [21] demonstrated that olanzapine, when used with other antiemetics, significantly reduced nausea and vomiting compared to placebo.

Overall, our analysis suggests that while olanzapine may offer a statistically significant benefit over aprepitant in controlling nausea during the overall phase of CINV, this advantage is not demonstrated in the acute or delayed phases. Moreover, the notable increase in somnolence with Olanzapine use necessitates a careful assessment of the risks and benefits when choosing an appropriate CINV prophylaxis, especially in patients where sedation may pose an increased risk or negatively impact quality of life. One limitation of this study is the exclusion of geriatric and pediatric age groups. Hence data pertaining to these groups were not included here. Another limitation was that the types of cancer as well as the treatment regimens varied across the eight studies, and as such may have resulted in differences in the resultant impact. 

## Conclusions

In conclusion, our study's comprehensive analysis indicates that though both olanzapine and aprepitant remain viable options or controlling CINV in the acute and delayed phases, olanzapine offers a statistically significant advantage in the control of overall phase nausea. This suggests that both medications can be effectively utilized for immediate and short-term CINV control. The consistency of these findings, even when accounting for study heterogeneity, reinforces the applicability of either drug in clinical settings during these phases. In practice, olanzapine tends to suit patients receiving highly emetogenic regimens-where even a slight reduction in breakthrough episodes improves adherence-and those who welcome gentle sedation, such as anxious, younger, or insomnia-prone individuals treated in outpatient settings with reliable caregiver support. By contrast, aprepitant (or another NK-1 antagonist) is often safer for elderly or frail patients at risk of falls, people who must drive or operate machinery, those already using CNS depressants, and individuals with metabolic syndrome who may be vulnerable to olanzapine’s longer-term weight-gain and glycemic effects. 

Conversely, the safety profile analysis revealed a considerably higher incidence of somnolence with olanzapine. Given the potential impact of such a side effect on patients’ quality of life and functional status, the risks and benefits should be carefully considered when prescribing olanzapine, particularly for patients where increased sedation is deemed unfavorable. Our findings support the notion that the choice of antiemetic medication must be individualized, taking into account both the efficacy in controlling CINV and the patient's tolerance and risk factors for adverse effects. An effort should be made to include patients in shared decision-making when choosing antiemetic therapy, actively weighing each option to formulate a final plan of treatment.
